# Gestational exposure to endocrine disrupting chemicals in relation to infant birth weight: a Bayesian analysis of the HOME Study

**DOI:** 10.1186/s12940-017-0332-3

**Published:** 2017-10-27

**Authors:** Meghan M. Woods, Bruce P. Lanphear, Joseph M. Braun, Lawrence C. McCandless

**Affiliations:** 1 Faculty of Health Sciences, Simon Fraser University, Blusson Hall, Rm 11300, 8888 University Drive, Burnaby, British Columbia V5A 1S6 Canada; 2 Child and Family Research Institute, BC Children’s and Women’s Hospital, 950 West 28th Avenue, Vancouver, British Columbia V5Z 4H4 Canada; 3 Department of Epidemiology, Brown University, Box G-S121-2, 121 South Main St, Providence, Rhode Island 02912 USA; 4 Department of Statistics and Actuarial Science, University of British Columbia, Faculty of Science, 3182 Earth Science Building, 2207 Main Mall, Vancouver, British Columbia V6T 1Z4 Canada

**Keywords:** Birth weight, Endocrine disruptors, Environmental exposure, Maternal exposure, Pregnancy

## Abstract

**Background:**

Pregnant women are exposed to a mixture of endocrine disrupting chemicals (EDCs). Gestational EDC exposures may be associated with changes in fetal growth that elevates the risk for poor health later in life, but few studies have examined the health effects of simultaneous exposure to multiple chemicals. This study aimed to examine the association of gestational exposure to five chemical classes of potential EDCs: phthalates and bisphenol A, perfluoroalkyl substances (PFAS), polychlorinated biphenyls (PCBs), polybrominated diphenyl ethers (PBDEs), and organochlorine pesticides (OCPs) with infant birth weight.

**Methods:**

Using data from the Health Outcomes and Measures of Environment (HOME) Study, we examined 272 pregnant women enrolled between 2003-2006. EDC concentrations were quantified in blood and urine samples collected at 16 and 26 weeks gestation. We used Bayesian Hierarchical Linear Models (BHLM) to examine the associations between newborn birth weight and 53 EDCs, 2 organochlorine pesticides (OPPs) and 2 heavy metals.

**Results:**

For a 10-fold increase in chemical concentration, the mean differences in birth weights (95% credible intervals (CI)) were 1 g (-20, 23) for phthalates, -11 g (-52, 34) for PFAS, 0.2 g (-9, 10) for PCBs, -4 g (-30, 22) for PBDEs, and 7 g (-25, 40) for OCPs.

**Conclusion:**

Gestational exposure to phthalates, PFAS, PCBs, PBDEs, OCPs or OPPs had null or small associations with birth weight. Gestational OPP, Pb, and PFAS exposures were most strongly associated with lower birth weight.

**Electronic supplementary material:**

The online version of this article (10.1186/s12940-017-0332-3) contains supplementary material, which is available to authorized users.

## Background

Endocrine disrupting chemicals (EDC) are synthetic compounds found in many consumer products [1–3] that alter the endocrine system [4, 5]. Because they are widely distributed in the environment, the majority of pregnant women in the United States have detectable levels of multiple EDCs in their blood or urine [6, 7]. Among EDCs, broad classes include phthalates and phenols (e.g., bisphenol A [BPA]), perfluoroalkyl substances (PFAS), polychlorinated biphenyls (PCBs), polybrominated diphenyl ethers (PBDEs), and organochlorine pesticides (OCPs). Phthalates are used as plasticizers [1, 4, 5, 8] and also found in personal care and consumer products [1, 3, 7]. BPA is added to many plastics and consumer products [9, 10]. PFAS are used as stain and water repellants [11], fire fighting foams [11] and food contact material [3] while PCBs were used as hydraulic oil additives [2], coolant fluids [2, 5] and insulators [12]. PBDEs are flame retardants [13] and OCPs are agricultural pesticides [5, 14].

The vulnerability of the developing fetus [15] coupled with the pervasive nature of EDCs has raised concerns about reproductive health effects arising from gestational EDC exposure, such as low birth weight [15]. Low birth weight is a predictor for coronary heart disease [16], diabetes mellitus [17] and infant mortality [17]. Additionally, some previously published studies [14, 15] found associations between gestational EDC exposure and birth weight. Understanding the role EDCs may play in affecting birth weight will inform efforts to prevent low birth weight and the development of chronic diseases.

Many investigators have explored the relationship between EDC exposures and birth weight [18]. Lenters et al. examined the relationships between 16 chemicals (6 phthalates, 8 PFAS, 2 PCBs and 1 OCP) and birth weight using Elastic Net Regression analyses; 2 phthalates, 1 PFAS, and 1 OCP were associated with lower birth weight [18]. Using a job exposure matrix, which can differ from exposure measurement, Birks et al. (2016) examined occupational EDC exposures in a meta-analysis of European birth cohorts and found that pregnant women exposed to more than 1 EDC class were more likely to have a low birth weight infant [15]. There is sufficient evidence that increased PFAS (especially PFOA) exposure is associated with low birth weight [18–20], but mixed results are reported for other EDCs and birth weight [18, 21]. For example, a meta-analysis by Govarts et al. (2016), found an association between PCB 153 and low birth weight [21] whereas Lenters et al. [18] did not find an association. Similar inconsistent associations exist for phthalates and BPA [22–24], OCPs [18, 25] and PBDEs [26, 27].

These inconsistencies may be due to a shortage of statistical tools to explore the effects of multiple chemical exposure mixtures on birth weight. One way to model multi-exposure mixtures is to collapse the exposures into a summary term that is included in regression models for the outcome [28] or use proxy variables [29]. These approaches, however, assign equal weight and importance to all chemicals, irrespective of their predictive value. Multivariable linear regression [5, 13, 24] with selected predictors [30] is another method, but it can produce unreliable estimates due to the highly correlated nature of some EDCs [31]. LASSO (Least Absolute Shrinkage and Selection Operator) [30] and Elastic Net are more sophisticated multivariable linear modeling methods that have been used for analyzing multiple correlated exposure variables.

Bayesian hierarchical linear models (BHLMs) are one possible solution to analyzing the effect of multiple correlated exposures [32]. BHLM is a statistical method belonging to a class of methods known as shrinkage methods that, by placing constraints on the distribution of the variables in the model, compress the beta coefficients toward a common mean, resulting in more stable estimates and avoiding the collinearity problem. This also reduces the Type I error (false positive) rate because the shrunken beta coefficients are closer to zero [33]. BHLM assumes that in a linear model, individual regression coefficients arise from a normal distribution, called the prior distribution. The prior distribution depends on hyperparameters, including a mean μ_β_ and standard deviation σ_β,_ which are informed by the data and can be quantified when fitted to the data. The quantity μ_β_ is the average association between all regression coefficients and the outcome, whereas σ_β_ captures the heterogeneity of effects.

The objective of this study was to use BHLM to simultaneously examine the association between exposure to 5 different EDC classes (phthalates and BPA, PFAS, PCBs, PBDEs and OCPs), 2 organophosphate pesticides (OPPs) and 2 heavy metals, lead (Pb) and mercury (Hg), with birth weight among women and their children in the Health Outcomes and Measures of Environment (HOME) Study [34].

## Methods

### HOME Study

The HOME Study is a prospective birth cohort study in the Cincinnati, Ohio area. Women were recruited between 13 and 19 weeks of pregnancy from prenatal clinics between 2003 and 2006. Inclusion criteria for the HOME Study included women who were >18 years old, <19 weeks gestation at the time of enrollment, and living in a residence built before 1987. Additional details about study eligibility, recruitment, and follow-up are described elsewhere [34]. The Cincinnati Children’s Hospital Medical Center (CCHMC) and participating delivery hospitals’ Institutional Review Boards (IRB) approved the HOME Study. After research assistants explained the study protocols, women provided consent for themselves and their children. Demographic information was collected via standardized questions administered by trained research assistants as described in more detail elsewhere [34] (Table 1).

**Table 1:** Distribution of birth weight in relation to participant characteristics among women in the HOME Study, 2003-2006, Cincinnati, OH

	*n* (%)	Birth weight (g) mean (SD)
All participants	384 (100%)	3371(618)
Age at delivery		
<25	93 (24.2%)	3079 (597)
25-<30	107 (27.8%)	3489 (876)
30-<35	121 (31.5%)	3469 (906)
>35	63 (16.5%)	3409(773)
Race		
White	273 (61.7%)	3514 (592)
Black or Other	147 (38.3%)	3141 (753)
Marital Status		
Living with spouse/partner	248 (64.5%)	3484 (600)
Not living with spouse/partner	136(35.5%)	3165 (746)
Employment Status		
Unemployed	74 (19.3%)	3278 (617)
Employed	310 (80.7%)	3392 (1263)
Income		
<25,000	124(32.3%)	3154 (729)
≥25,000	260 (67.7%)	3475 (600)
Insurance		
Private	272 (70.8%)	3466 (601)
Public/None	112(29.2%)	3141 (714)
Education Level		
≥High school	343 (89.3%)	3409 (608)
< High school	41(10.7%)	3050 (644)
Tobacco Use (Mean Cotinine Levels)		
Non smoker (≤ 3 ng/ml)	341(88.8%)	3387 (617)
Active Smoker (> 3 ng/ml)	43 (12.1%)	3241 (655)
Gender of Baby		
Male	178 (46.3%)	3477 (610)
Female	206 (53.7%)	3279 (897)
BMI (kg/m^2^)		
Underweight	4 (0.1%)	2940 (625)
Normal	159 (41.4%)	3328 (618)
Overweight	127 (33.1%)	3412 (828)
Obese	94 (24.4%)	3406 (779)

Abbreviations: *SD* standard deviation, *BMI* body mass index

### Outcome variables

The outcome variable was birth weight, measured in grams (g). Birth weight was abstracted from birth records and analyzed as a continuous variable.

### Biomarkers of gestational EDC exposure

Blood and urine samples were collected from participants at approximately 16 weeks and 26 weeks gestation. We used sensitive and specific liquid or gas chromatography mass spectrometry [34] to measure gestational EDC exposure. A total of nine phthalates, BPA, 5 PFAS, 23 PCBs, 9 PBDEs, 6 OCPs, 2 OPPs and 2 heavy metals were measured (Table 2). EDCs with measurements below the limit of detection (LOD) were assigned LOD/√2 [10, 35, 36]. Phthalate metabolites and BPA were creatinine-normalized in μg/g creatinine and measured twice. The metals were also measured twice while remaining biomarkers were only measured once. PFAS were expressed in units of μg/L serum. PCBs, PBDEs, OCPs and OPPs were lipid-normalized in units of ng/g serum lipid. All biomarker samples were log_10_ transformed and treated as continuous variables. If a woman provided multiple urine or blood samples at both 16 and 26 weeks gestation, then the sample concentrations were averaged following log_10_ transformation and treated as continuous variables [34, 37]. Biomarkers with <90% detection level or that were only examined in a subset of the participants were excluded from analysis to preserve sample size (Table 2). Building on a previous analysis of the HOME Study data [14], we examined OPPs as the molar sum of diethylphosphate (DEP) and dimethylphosphate (DMP) metabolite concentrations. Pb, while not conventionally considered an EDC, is a widespread reproductive [38] toxin that impacts neurodevelopment [6] and birth weight at very low exposure levels [39] and so is included in this study.

**Table 2:** Concentrations (μg/g creatinine phthalates and BPA, ng/g lipids PCBs, PBDEs and OCPs, μg/L serum PFAS, DEP, DMP and Hg, μg/dL Pb) of EDCs collected via urine or blood sample from pregnant women for HOME Study, 2003-2006, Cincinnati, OH, *n*=272

				Percentiles			Included in Analyses	Median NHANES (2003-2004)
EDC	%>LOD	Mean LOD^b^	GM (GSD)	25th	50th	75th		
MBP	100	0.60	27.2 (1.9)	17.6	25.8	37.8	Yes	24
MIBP	99	0.30	5.9 (1.9)	4.0	6.1	9.1	Yes	4.0
MEP	100	0.53	152 (3.0)	77.4	143	330.7	Yes	120
MBZP	98	0.22	9.8 (2.4)	5.6	10.7	14.9	Yes	10
MCPP	99	0.20	2.4 (1.9)	1.6	2.3	3.4	Yes	2.9
MEHP	98	1.20	5.9 (2.8)	3.3	4.7	10.4	Yes	2.2
MECPP	100	0.60	41.3 (2.5)	21.5	34.9	70	Yes	31
MEHHP	100	0.70	28.7 (2.6)	14.9	25.3	53.2	Yes	19
MEOHP	100	0.70	22.3 (2.5)	12.3	18	40.3	Yes	^a^
BPA	96	0.4	2.1(1.9)	1.4	1.9	2.9	Yes	2.7
PCB-28	82	0.79	0.7 (3.1)	0.6	1	1.4	No	5.0
PCB-44	92	0.53	0.07 (1.8)	0.04	0.06	0.09	Yes	^a^
PCB-49	100	0.53	0.06 (1.8)	0.04	0.05	0.07	Yes	^a^
PCB-52	91	0.53	0.1 (3.1)	0.04	0.07	0.2	Yes	^a^
PCB-66	75	0.53	0.4 (3.7)	0.1	0.6	1	No	1.4
PCB-74	99	0.63	2.8 (1.8)	1.9	2.7	4	Yes	5.4
PCB-87	100	0.53	0.09 (2.9)	0.04	0.06	0.2	Yes	^a^
PCB-99	99	0.53	2.7 (2.1)	1.7	2.8	3.9	Yes	3.9
PCB-101	32	0.53	0.1 (2.1)	0.05	0.07	0.4	No	1.6
PCB-105	93	0.53	1 (2.8)	0.6	1.1	1.7	Yes	1.2
PCB-110	99	0.53	0.09 (3)	0.04	0.06	0.1	Yes	^a^
PCB-118	99	0.78	5 (1.9)	3.1	4.8	7	Yes	5.0
PCB-128	97	0.53	0.07 (2.5)	0.04	0.05	0.08	Yes	^a^
PCB-146	93	0.53	1 (2.9)	0.8	1.1	1.8	Yes	2.3
PCB-149	97	0.53	0.08 (2.5)	0.04	0.06	0.1	Yes	^a^
PCB-151	97	0.53	0.07 (2.2)	0.04	0.05	0.08	Yes	^a^
PCB-153	100	0.81	11.4 (1.9)	7.5	10.9	15.3	Yes	22
PCB-156	96	0.53	1.6 (2.4)	1	1.6	2.7	Yes	3.4
PCB-157	51	0.53	0.2 (3.7)	0.06	0.2	0.6	No	0.9
PCB-167	59	0.53	0.2 (3.9)	0.06	0.3	0.6	No	0.9
PCB-170	100	0.61	2.9 (2.2)	1.8	2.8	4.3	Yes	6.3
PCB-172	34	0.53	0.1 (3.5)	0.05	0.08	0.4	No	0.9
PCB-177	61	0.53	0.2 (4.1)	0.07	0.4	0.7	No	1.3
PCB-178	52	0.53	0.2 (4)	0.06	0.2	0.7	No	1.2
PCB-180	100	0.78	6.7 (2.1)	4.1	6.3	9.7	Yes	^a^
PCB-183	87	0.53	0.7 (3.3)	0.5	0.8	1.4	Yes	1.7
PCB-187	98	0.53	2 (2.4)	1.3	2	3.2	Yes	4.6
PCB-189	97	0.53	0.07 (2.1)	0.04	0.05	0.08	Yes	^a^
PCB-194	92	0.53	1.3 (2.7)	0.9	1.4	2.2	Yes	4.0
PCB-195	39	0.53	0.1 (3.5)	0.05	0.08	0.5	No	0.6
PCB-199	93	0.53	1.2 (2.9)	0.8	1.3	2.2	Yes	3.7
PCB-206	81	0.53	0.9 (3.5)	0.4	0.9	1.3	Yes	2.3
PCB-209	35	0.53	0.1 (3.2)	0.05	0.08	0.4	No	1.2
PCB-38/158	99	0.78	8 (2.1)	5.2	7.8	11.3	No	16
PCB-196/203	96	0.53	1.5 (2.4)	1.1	1.6	2.6	No	3.3
PFOA	100	6.05	5.6 (1.7)	3.8	5.4	8.1	Yes	3.6
PFOS	100	14.2	13.6 (1.5)	10	14.4	17.9	Yes	18
PFNA	100	0.96	1 (1.4)	0.8	1	1.2	Yes	0.9
PFHXS	100	1.96	1.6 (2)	1	1.6	2.6	Yes	1.6
PFDEA	30	0.21	0.2 (1.6)	0.2	0.2	0.3	Yes	^a^
BB-153	85	0.53	0.9 (4.1)	0.5	1.1	2	No	^a^
PBDE-17	96	0.53	0.07 (2.2)	0.04	0.06	0.08	Yes	^a^
PBDE-28	81	0.53	0.7 (4.1)	0.3	0.9	1.7	Yes	1.0
PBDE-47	100	0.86	19.1 (2.7)	9.6	18	30	Yes	19
PBDE-66	92	0.53	0.06 (2)	0.04	0.05	0.07	Yes	^a^
PBDE-85	49	0.53	0.2 (4.8)	0.06	0.2	0.7	Yes	<LOD
PBDE-99	100	0.65	4.4 (2.9)	2.2	4.2	7.4	Yes	<LOD
PBDE-100	99	0.53	3.7 (3)	2	3.5	6.4	Yes	3.2
PBDE-153	99	0.53	5.2 (3.3)	2.4	4.2	9.2	Yes	4.0
PBDE-154	42	0.53	0.2 (4.7)	0.05	0.1	0.6	No	0.8
PBDE-183	8	0.53	0.1 (3.3)	0.04	0.07	0.2	Yes	^a^
β-HCH	27	2.63	0.5 (3.5)	0.22	0.32	1.6	Yes	<LOD
HCB	94	3.92	6.6 (1.9)	5.5	7.2	9.1	Yes	16
p'p'-DDT	52	2.63	1.1 (3.7)	0.32	1.9	3.2	Yes	<LOD
p'p'-DDE	100	2.63	74 (1.8)	51	70	102	Yes	206
Oxychlordane	90	2.63	4.4 (2.5)	3.4	5.1	7.5	Yes	11
*trans-*Nonachlor	97	2.63	7.5 (2.1)	5.1	7.5	12	Yes	15
DEP	8	0.56	0.08 (5.4)	0.02	0.09	0.3	Yes	^a^
DMP	27	0.62	0.4 (4.2)	0.1	0.4	0.9	Yes	^a^
Pb	96	0.25	0.7 (1.4)	0.5	0.7	0.8	Yes	^a^
Hg	79	0.2	0.6 (2.3)	0.4	0.6	1.0	Yes	^a^

Abbreviations: *GM* geometric mean, *GSD* geometric standard deviation, *BPA* bisphenol A, *β-HCH* beta-hexachlorocyclohexane, *HCB* Hexachlorobenzene, *DDT* dichlorodiphenoltrichloroethane, *DDE* dichlorodiphenoldichloroethylene

^a^Data not available. ^b^LODs varied by sample size; LODs for PCBs, PBDEs and OCPs are lipid-adjusted and measured in pg/g

### Potential Confounders

We drew a directed acyclic graph to select confounders based on the relationships between the HOME Study [10, 34] covariates and EDCs and birth weight (Additional file 1: Figure S1). Covariates in the birth weight BHLM included: maternal race (white vs. black/other), age at delivery, infant sex, maternal education (<high school vs. ≥high school), tobacco exposure (<3ng/ml vs. ≥3ng/ml serum cotinine concentrations), household annual income (<$25,000 per annum vs. >$25,000 per annum), employment, maternal insurance status (public/none vs. private), marital status (single vs. living with a spouse or partner), pre-natal vitamin use (yes vs. no), and maternal BMI (underweight, normal, overweight, obese). To account for the effect of gestation duration on birth weight, we controlled for gestational age (GA) non-parametrically using the 'splines' package in R [40].

### Statistical Analyses

We used descriptive analyses to examine the distribution of EDC variables, birth weight, and participant characteristics. We calculated basic descriptive statistics for participant variables (Table 1), EDC concentration geometric means and percentiles (Table 2). We also examined pair wise EDC correlation in a heat map (Additional file 1: Figure S2). In multivariable modeling, each EDC exposure was standardized (mean = 0, standard deviation = 1) and BHLM was fitted using Bayesian software Stan and the 'rstan' package in R [40, 41]. The final adjusted model was: Y = β_0_ + βx_1_X_1_....βx_p_X_p_ +βc_1_C_1_+....+βc_k_C_k_ +spline(GA)+ error, where Y is the outcome birth weight, β_0_ is the y-intercept, βx_1_...βx_p_ are the exposure variable regression coefficients, X_1_...X_p_ are the exposure variables, spline(GA) is the non-parametric spline for GA, and C_1_…C_k_ are the 9 covariates. All 53 exposure variables from 5 chemical classes were included in the final BHLM model for analysis. BHLM assumes that βx_i_ are Gaussian random effects with group specific mean μ_β_ and standard deviation σ_β_. The hyperparameter μ_β_ is the average association between the exposure variables within a chemical class and the outcome, whereas σ_β_ governs the heterogeneity of effects for the class regression coefficients. The hyperparameters for the different chemical classes were assigned uninformative prior distributions: μ_β_ normal with mean 0 and variance 10^5^, and σ_β_ normal with mean 0 with variance 10^5^ such that σ_β_>0. Hamilton Monte Carlo sampling was used to obtain 2 chains of 20,000 iterations with 500 warm-up iterations. The 2 OPPs, Pb and Hg were included in the model as covariates without hierarchical modeling.

The final results included both 95% and 50% equi-tailed posterior credible intervals (95%, 50% CI) for parameter estimates. The 50% CI is the smallest interval that contains 50% posterior probability of containing the true parameter. Using 50% CIs gives higher power to detect small effects in the data and they can serve as a screening tool for identifying weak effects that may warrant further study [33]. Reporting the 50% CIs also decreases the Type II error resulting from BHLM shrinkage. However, a limitation of 50% CIs when used for multiple frequentist hypothesis tests is that they have a higher Type I error rate than using alpha level = 0.05.

Additionally, to better understand the impact of the BHLM shrinkage on the regression coefficients, we re-analyzed the data using both LASSO and Elastic Net. We used the R package ‘glmnet’ [42], and included the BHLM confounders (Table 3) in the regression as unpenalized variables. Cross-validation was used to determine the penalization parameter. Note that confidence intervals for beta coefficients from LASSO and Elastic Net are not available using standard software, and calculating standard errors for parameter estimates is challenging because the shrinkage estimation produces biased estimates for large coefficients. Following the approach of Lenters et al. [18], the predictors selected by LASSO and Elastic Net were incorporated into an omnibus multiple linear regression model to obtain unpenalized, mutually adjusted estimates.

**Table 3:** Bayesian estimates of the average of the beta coefficients, denoted μ_β_, within each EDC class in relation to birth weight (g) in the HOME study, 2003-2006, n=272, Cincinnati, OH

	Difference in birthweight (grams)					
	Primary analysis (GA Included)			Secondary analysis (GA Excluded)		
Independent variable	Posterior mean (SD)	95% CI	50% CI	Posterior mean (SD)	95% CI	50% CI
Phthalate Class and BPA	1.18(10.3)	(-19.8, 23.4)	(-4.28, 6.34)	2.66(10.4)	(-18.4, 22.4)	(-3.32, 8.85)
PFAS Class	-10.6(23.8)	(-51.5, 34.0)	(*-20.9*, *-2.26*)	-18.7(28.0)	(-72.6, 36.2)	(*-30.5*, *-6.99*)
PCB Class	0.22(4.70)	(-9.31, 10.1)	(-2.48, 2.91)	0.47(6.21)	(-11.3, 13.6)	(-3.04, 3.37)
PDBE Class	-3.93(12.5)	(-30.8, 22.2)	(-9.94, 2.27)	-9.07(15.1)	(-37.4, 22.6)	(*-16.6*, *-2.29*)
OC Pesticide Class	7.26(17.9)	(-25.4, 39.9)	(-2.63, 16.1)	13.3(22.1)	(-28.5, 56.5)	(1.61, 25.1)
DEP	14.1 (33.2)	(-51.4, 79.3)	(-8.02, 35.9)	-18.6(43.4)	(-103, 64.6)	(-48.1, 11.5)
DMP	-56.8(33.8)	(-123, 10.2)	(*-79.3*, *-34.0*)	-69.4(45.2)	(-156, 20.8)	(*-101*, *-38.8*)
Lead	-44.8(33.5)	(-110, 21.7)	(*-67.6*, *-22.2*)	1.51(44.4)	(-83.9, 88.2)	(-27.9, 31.4)
Mercury	5.44(31.2)	(-55.9, 66.4)	(-15.7, 26.6)	-13.9(39.7)	(-92.6, 63.7)	(-40.8, 12.7)
Race (Black/Other)	-98.2(90.6)	(-277, 82.7)	*(-158*, *-38.6*)	-274(119)	(*-508*, *-37.6*)	(*-354*, *-193*)
Age at Delivery	6.04(7.71)	(-8.95, 21.1)	(0.83, 11.3)	1.48(9.97)	(-18.2, 20.8)	(-5.24, 8.31)
Infant Sex (female)	-201(58.0)	(*-315*, *-87.8*)	(*-240*, *-162*)	-163(72.9)	(*-307*, *-21.1*)	(*-212*, *-114*)
Did not finish high school	-124(80.1)	(-282, 34.2)	(*-177*, *-70.4*)	-98.2(104)	(-302, 104)	(-168, -28.1)
Cotinine (ng/ml >3)	-76.7(38.2)	(-151, 0.53)	(*-103*, *-51.3*)	-98.8(49.5)	(-195, -2.45)	(*-133*, *-65.8*)
Low income (<$25,000/yr)	-35.7(102)	(-237, 163)	(-104, 34.8)	86.3(131)	(-173, 344)	(-1.91, 173)
Unemployment	-83.6(76.7)	(-233, 65.9)	(*-135*, *-31.0*)	-5.46(101)	(-205, 189)	(-73.2, 64.0)
Public/No Insurance	91.4(106)	(-117, 301)	(19.1, 162)	15.0(141)	(-251, 291)	(-80.1, 109)
Marital Status (Unmarried)	-67.2 (99.8)	(-263, 132)	(*-134*, *-1.78*)	-68.4(143)	(-364, 208)	(-161, 27.2)
Pre-natal vitamins (Yes)	139(68.2)	(273, 6.99)	(185, 92.2)	92.9(88.1)	(266, 81.1)	(150, 33.4)
BMI (kg/m^2^)	19.6(4.96)	(9.84, 29.2)	(16.2, 23.0)	23.8(6.28)	(11.9, 36.3)	(19.4, 28.0)

Abbreviations: *SD* standard deviation, *CI* credible interval, *SES* socioeconomic status, *BMI* body mass index

Significance: Italicized 95% CIs and 50% CIs do not contain zero and therefore are considered significant at *p* = 0.05 or *p* = 0.5 respectively

## Results

Of 389 singleton live births, 272 (69.9%) had all covariates and were included in analysis. The study participants were mostly white (61%), married (64%), and university educated (50%) (Table 1). Several maternal demographic characteristics predicted birth weight, including age, race, marital status, annual household income, public or no insurance and other SES variables. Tobacco smoke exposure also predicted lower birth weight with a 210 g reduction among smoking mothers (>3 ng/ml serum cotinine) compared with non-smoking mothers (<3 ng/ml serum cotinine).

With few exceptions (e.g., PFOA), median concentrations among HOME Study participants tended to be similar to concentrations observed in US women (Table 2). The GM of phthalate and BPA concentrations ranged from 2.4 ng/ml (MCPP) to 41.3 ng/ml (MECPP). The PFAS exhibited a minimum GM of 1 μg/L (PFNA) and maximum GM of 13.6 μg/L (PFOS). PCBs ranged from 0.06 ng/ml (PCB 49) to 11.4 ng/ml (PCB 153), PBDEs held a minimum GM 0.06 ng/ml (PBDE 66) and a maximum concentration of 19.1 ng/ml (PBDE 47). The lowest OCP GM was DDT (1.1 ng/ml), and DDE had the highest GM (74 ng/ml). Individual chemicals within a class tended to be positively correlated with each other, particularly for persistent compounds like PCBs and PBDEs (Additional file 1: Figure S2 [43]). Phthalates and BPA were less strongly correlated with other chemicals and, with the exception of the di-2-ethylhexylphthalate metabolites, weakly correlated with each other.

The phthalates and BPA, PFAS, PCBs, PBDEs, OCPs, OPPs, Pb and Hg were examined using BLHM (Table 3). The μ_β_ posterior mean for the phthalates and BPA was 1 g with 95% CI (-20, 23), meaning the average association between the 9 phthalates and BPA and birth weight was 1 g. Another way to interpret this is that for every 10-fold increase in exposure to phthalates and BPA as a whole, there was a 1 g increase in birth weight. This increase was not statistically significant at the 0.05 level based on a frequentist hypothesis test using the posterior credible interval. The σ_β_ posterior mean was 10 g, which indicated heterogeneity in the phthalate and BPA effects around this estimate, and that roughly two thirds of the beta coefficients were within 10 g (one standard deviation) of μ_β_. In contrast, for PFAS exposure, the μ_β_ and σ_β_ posterior means were larger in magnitude and equal to -11 g, 95% CI (-52, 34) and 24 g, respectively. Although the 95% CI for μ_β_ for PFAS did cover zero, the estimate was significantly lower than zero based on the upper limit of the 50% CI (-21, -2). For PCB exposure, the μ_β_ posterior mean was 0.2 g, 95% CI (-9, 10). The PBDE μ_β_ estimate was -4 g, 95% CI (-31, 22). Finally, OCP exposure was associated with a μ_β_ of 7 g, 95% CI (-25, 99) with an estimated σ_β_ of 18 g.

**Fig. 1:**
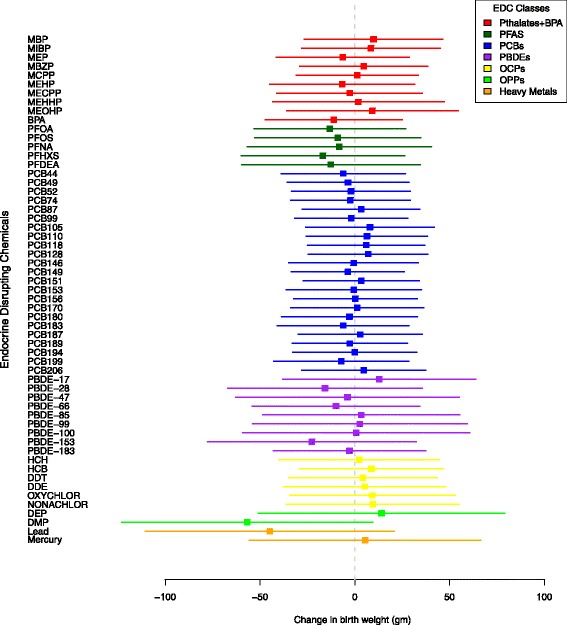
Posterior 95% CIs of BHLM regression coefficients, HOME Study, 2003-2006, n=272, Cincinnati, OH

**Fig. 2:**
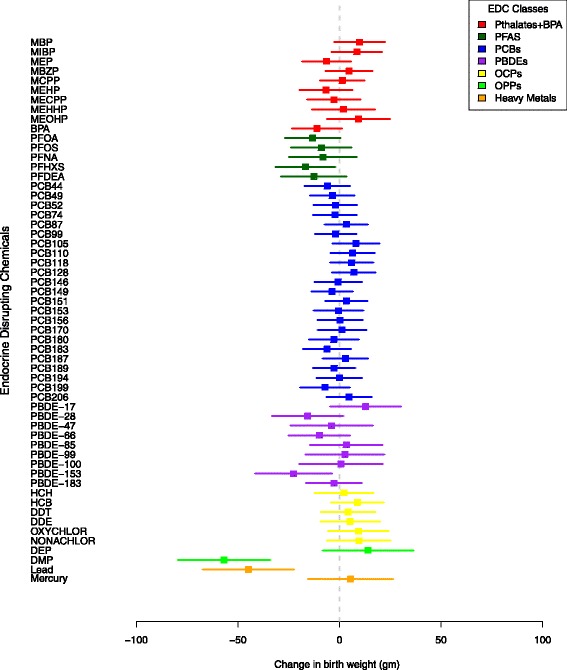
Posterior 50% CIs of BHLM regression coefficients, HOME Study, 2003-2006, n=272, Cincinnati, OH

We examined associations between individual EDCs and birth weight to understand which individual EDCs within a class drive the value of μ_β_, given by the posterior distribution of the BHLM regression coefficients βx_1_ ... βx_p_ (see Figs. 1 and 2). In the phthalates and BPA class, there was a 9 g birth weight increase in response to a 10-fold increase exposure to MEOHP, and a 10-fold increase in MEHP exposure was associated with a 7 g decrease in birth weight. None of the individual phthalate metabolites or BPA were statistically significant at the 0.05 or 0.5 level, which may explain why the class as a whole was not significantly different from zero. In contrast, all 5 PFAS were associated with reductions in birth weight, although none of the 95% CIs excluded zero. PFHXS exhibited the largest association in the BHLM with a 10-fold exposure increase associated with a 17 g birth weight decrease, whereas PFNA was the weakest predictor of birth weight. PCBs ranged from an 8 g birth weight increase associated with a 10-fold increase of PCB 105 to a 7 g decrease associated with a 10-fold increase in PCB 199. The large number of 23 PCBs examined in this study meant that the BHLM performed more aggressive shrinkage on the model regression coefficients for this class. Of the 9 PBDEs, a 10-fold increase in PBDE 17 was associated with the greatest birth weight increase (12 g) while PBDE 153 was associated with the greatest decrease (23 g). The largest increase in the OCP class was seen with a 10-fold increase in HCH exposure (5 g), while the largest birth weight decrease was seen in response to a 10-fold increase in Nonachlor exposure (10 g). None of the OCP class 95% CIs excluded zero. The two OPPs and two heavy metals were examined individually as covariates with no hierarchical priors. For every 10-fold increase in DEP exposure, there was a 14 g increase in birth weight 95% CI (-51, 79). DMP exposure was associated with a 57 g reduction in birth weight (95% CI (-123, 10), 50% CI (-79, -34)). Blood lead concentration was associated with a 45 g decrease in birth weight (95% CI (-110, 22), 50% CI (-68, -22)), while mercury exposure was associated with a 5 g, 95%CI (-56, 66) increase in birth weight.

To examine the impact of shrinkage and the choice of prior distributions on the BHLM results, we applied LASSO and Elastic Net to the phthalates and BPA,PFAS, PCBs, PBDEs and OCPs. As with the BHLM, Pb, Hg and OPPs were included as covariates. LASSO selected 7 predictor variables (2 phthalates, 2 PCBs, 2 PBDEs and 1 OCP) out of a possible 53. The LASSO results are given in Additional file 1: Figure S3. The LASSO regression coefficients tended to be larger in magnitude than BHLM with wider and less precise 95% intervals, which we expected because LASSO does not use shrinkage that borrows information between chemicals within a particular class. Elastic Net selected the same variables as LASSO, as well as an additional 5 PCBs and 1 PBDE. The results are given in Additional file 1: Figure S4. Our Elastic Net analysis identified 1 EDC in common with Lenters et al. [18], the phthalate MEOHP. In Lenters, this EDC was associated with a -0.15 g difference in birth weight, which was attenuated after adjusting for gestational age. In our analysis, MEOHP was associated with a 13 g birth weight increase (Additional file 1: Figure S3). Additionally, Pb was associated with a 58 g decrease in birth weight in the LASSO analysis (Additional file 1: Figure S3) and a 53 g decrease in the Elastic Net analysis (Additional file 1: Figure S4). DEP, one OPP, was associated with a 16 g birth weight increase using LASSO (Additional file 1: Figure S3) and DMP, the other OPP, was associated with a 48 g decrease (Additional file 1: Figure S3). In the Elastic Net analysis, DEP was associated with a 21 g increase and DMP with a 54g decrease in birth weight (Additional file 1: Figure S4). Because LASSO and Elastic Net do not estimate the μ_β_ parameter, this makes Additional file 1: Figures S2 and S3 harder to interpret because they do not estimate the average association between the exposure variables and the outcome within a chemical class.

GA is a potential mediating variable on the causal pathway between EDC exposure and birth weight, while sex is a potential modifier. Controlling for mediators may attenuate the total effect of EDCs on birth weight. Accordingly, we conducted sensitivity analyses without controlling for GA (Table 3) and stratified the BHLM results by sex (Additional file 1: Tables S1 and S2). While the results are mostly unchanged, the phthalates and BPA exposures were associated with a decrease in birth weight for male infants compared to the combined analysis (3 g decrease compared with 1 g increase). The decrease in male birth weight associated with Pb exposure was roughly double the associated birth weight decrease in the combined analysis (94 g decrease compared with 45 g decrease). There are some potentially interesting differences in the impact of DEP and DMP on birth weight when the study sample is stratified by infant sex. While DEP was associated with a 14 g increase in birth weight in the combined analysis, it was associated with a 51 g increase in males and a 9 g decrease in females in the sex stratified analysis. DMP in the combined analysis was associated with a 57 g decrease in birth weight; looking at the stratified analysis revealed that this decrease is greater in magnitude for male infants (88 g decrease), and lesser in female infants (27 g decrease). The associations of greater magnitude present in male infants correspond with previously published studies [24].

## Discussion

In this study we examined the relationship between gestational exposure to 53 different EDCs and birth weight in a prospective birth cohort of mother-infant pairs. We found that exposure to phthalates and BPA, PFAS, PCBs, PBDEs or OCPs had null or small associations with birth weight. Other investigators have reported similar small or statistically non-significant associations between birth weight and phthalates [37], PCBs [15], PBDEs [13], and OCPs [25]. Several other studies, however, have found associations between increased exposure to phthalates, PCBs, PBDEs or OCPs whereas we did not. A case-control study by Zhang et al. [24] revealed that low birth weight infants in China tended to have much higher meconium phthalate levels than infants who were normal weight. Additionally, a meta-analysis by Govarts et al. [21] reported that increased exposure to PCBs was associated with decreased birth weight. Lignell et al. [27] found a statistically significant inverse association between PBDE exposure and birth weight. Finally, Monteagudo et al. [44] and Lenters et al. [18] both found changes in birth weight associated with increased OCP exposure.

We found some evidence that PFAS, DMP and Pb were associated with small reductions in birth weight. On average, a 10-fold increase in exposure to the PFAS class was associated with an -11 g change in birth weight, 50% CI (-21, -2). However, due to the higher Type I error rate associated with using alpha level = 0.5, this association should be interpreted with caution. Similar findings concerning PFAS exposure have been reported elsewhere [5, 19]. While the exact mechanism by which PFAS impact birth weight is unknown, several theories have been proposed. One explanation is that PFAS interact with estrogen receptors and disrupt hormonal balances [20]. Additionally, PFAS alter human [20] and animal [3] serum lipid levels, potentially affecting fetal growth and development. Finally, PFAS affect adipose tissue development and the regulatory systems involved in body weight homeostasis, which may impact fetal growth outcomes [20], including those that occur after birth such as infant mortality or cardiovascular disease which are beyond the scope of this study [16, 17]. A 10-fold increase in Pb exposure was associated with a 45 g birth weight reduction (50% CI (-68, -22), while increased exposure to DMP was associated with a -57 g (50% CI(-79, -34)) change in birth weight. Both of these findings, as with the PFAS findings, should be interpreted cautiously because alpha = 0.5. They do, however, agree with previously published studies [14, 39, 45, 46].

The BHLM approach has advantages compared with other analytic techniques including LASSO and Elastic Net. First, each EDC class contains between 4 to 23 chemicals with varying associations between any given chemical and birth weight. Having many exposure variables complicates the interpretation of associations between the 5 EDC classes and birth weight when using traditional multi-variable linear methods. However, BHLM examines the average effect of EDC exposure on birth weight at the class level using the parameter μ_β_, which is less susceptible to random variation [32]. A second advantage of reporting μ_β_ is that it prevents the investigator from only reporting chemicals that are significantly related to birth weight by virtue of it being an average association between a class and the outcome. For example, rather than sorting through 23 PCBs to identify those that are significant, we simply reported the average association of all 23 PCBs and birth weight. Additionally, Bayesian modeling produces the σ_β_ value that measures the heterogeneity of EDC class effects. A final advantage of Bayesian modeling is that posterior distributions of the regression coefficients are shrunk towards pooled mean μ_β_, reducing the Type I error (false positives) rate when the interval estimates are used for frequentist hypothesis testing.

There were several limitations to the study. First, the BHLM model did not account for interactions between classes, and we assumed that all chemicals within a class share a similar mode of action and arise from a common prior distribution. Second, the model also excluded non-linear relationships, including potential threshold effects that could yield important information about the relationships between EDC exposure and birth weight. Third, BHLM results are sensitive to the assigned prior distributions of hyperparameters μ_β_ and σ_β_. We used priors following recommendations of a recent study of vague priors for random effects models [47]. BHLM, because it is a shrinkage method, is also prone to higher Type II errors. We included the 50% CIs in our study to account for this. Fourth, our study includes both persistent and non-persistent EDCs when examining EDC exposure. Non-persistent chemicals exhibit larger degrees of measurement error. For example, phthalates metabolites and BPA have short half-lives and this may have attenuated regression estimates and reduced statistical power [48]. To reduce exposure misclassification, we pooled the exposures at 16 and 26 weeks [40]. Fifth, we adjusted for gestational age non-parametrically. Other methods to adjust for gestational age include birth weight z-scores [49] and semi-parametric modeling [50]. Lastly, we conducted these analyses under the assumption that structurally similar chemicals would have similar modes of action and therefore should be grouped together. Other grouping schemes, however, may be more informative.

## Conclusion

This paper introduces BHLM as a method for tackling multiple correlated exposures in relation to health outcomes and compared this method with LASSO and Elastic Net. LASSO and Elastic Net regression coefficients were larger in size but with less precise 95% intervals compared to BHLM. BHLM uses shrinkage estimation to bias the regression coefficient estimates towards the group mean parameter. Statistical theory demonstrates that the estimators have smaller mean squared error than ordinary least squares estimators [51]. BHLM also has the advantage that it yields estimates of the hyperparameters that aid in interpreting the effects of multiple correlated exposures. We showed that gestational exposure to phthalates and BPA, PCBs, PBDEs or OCPs had small or null associations with differences in birth weight. In contrast, and consistent with previous studies, PFAS, Pb, and OPP exposures were more strongly associated with reduced birth weight [19, 39, 45, 46].

## Additional file

Additional file 1: Additional data visualizations including DAG, correlation heat map, and LASSO and Elastic Net figures and tables for secondary sex-stratified analysis. (DOCX 331 kb)

## Abbreviations

BHLM: Bayesian Hierarchical Linear Models
BPA: bisphenol A
CI: Credible interval
EDC: Endocrine disrupting chemicals
GA: Gestational age
GM: Geometric mean
Hg: Mercury
HOME: Health Outcomes and Measures of Environment
LASSO: Least Absolute Shrinkage and Selection Operator
LOD: Limit of detection
OCP: Organochlorine pesticides
OPP: Organophosphate pesticides
Pb: Lead
PBDE: Polybrominated diphenyl ethers
PCBs: Polychlorinated biphenyls
PFAS: Perfluoroalkyl substances
SES: Socioeconomic status
