# Supplementary material for: Gestational exposure to endocrine disrupting chemicals in relation to infant birth weight: a Bayesian analysis of the HOME Study
Source: Environ Health. 2017 Oct 27;16:115. doi: 10.1186/s12940-017-0332-3 (PMC5658906; doi:10.1186/s12940-017-0332-3)
Supplement: Additional file 1: — Additional data visualizations including DAG, correlation heat map, and LASSO and Elastic Net figures and tables for secondary sex-stratified analysis. (DOCX 331 kb) [file 12940_2017_332_MOESM1_ESM.docx]

Supplementary materials for ENHE-D-17-00050

Gestational exposure to endocrine disrupting chemicals in relation to infant birth weight: A Bayesian analysis of the HOME Study

by Woods MM, Lanphear BP, Braun JM and McCandless LC

Figure S1. Directed acyclic graph for relationship between EDC exposure, participant variables and birth weight. The unknown variable U represents existing maternal metabolic derangements associated with metabolism and absorption distributions, which would affect the metabolism and excretion of EDCs, as well as maternal BMI. .............................................................................................................................pg2

Figure S2. Heat Map of correlation coefficients between EDCs in HOME Study, n=272, Cincinnati, OH, 2003-2006................................................................................................................................................pg 3

Table S1. Bayesian estimates of the average of the beta coefficients, denoted μ_β_, within each EDC class in relation to birth weight (g)of 125 male infants in the HOME study, 2003-2006, Cincinnati, OH .......................................................................................................................................................pg 4

Table S2. Bayesian estimates of the average of the beta coefficients, denoted μ_β_, within each EDC class in relation to birth weight (g) of 147 female infants in the HOME study, 2003-2006, Cincinnati, OH............................................................................................................................................................................pg 6

Figure S3. 95% CIs for beta coefficients estimated using LASSO, HOME Study, 2003-2006, n=272, Cincinnati, OH. Predictor variables that were dropped from the model by LASSO are denoted by point estimates at zero............................................................................................................................pg 8

Figure S4. 95% CIs for beta coefficients estimated using Elastic Net, HOME Study, 2003-2006, n=272, Cincinnati, OH. Predictor variables that were dropped from the model by Elastic Net are denoted by point estimate at zero....................................................................................................................pg 9

**Figure S1.**  Directed acyclic graph for relationship between EDC exposure, participant variables and birth weight. The unknown U variable represents existing maternal metabolic derangements associated with metabolism and absorption distributions, which would affect the metabolism and excretion of EDCs, as well as maternal BMI. The biomarker measurement variable serves as a reminder that actual exposures to EDCs might be different from the measured concentration due to pharmacokinetics, which is influenced by variables like unknown variable U and BMI. This, while not the focus of this study, is important for the conceptual formation of measures of EDC exposure.


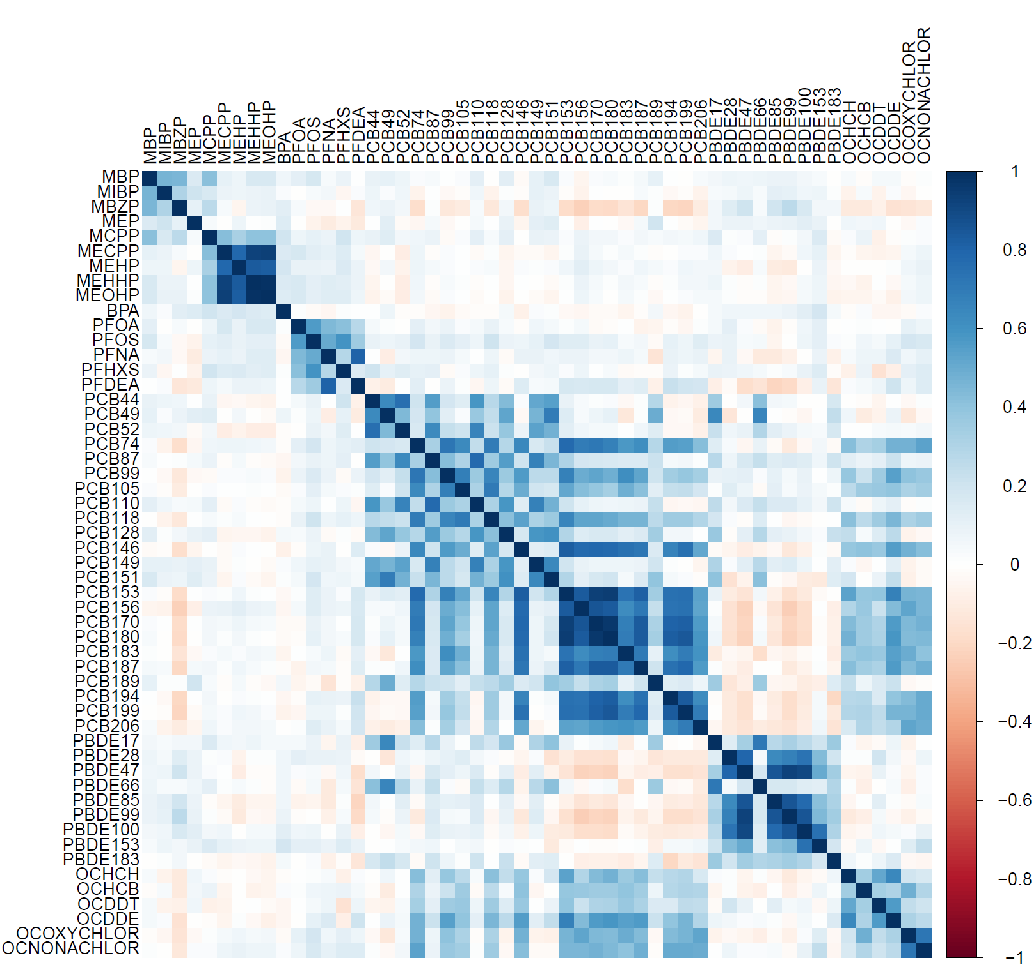


**Figure S2.**Heat Map of correlation coefficients between EDCs in HOME Study, n=272, Cincinnati, OH, 2003-2006.

**Table S1.** Bayesian estimates of the average of the beta coefficients, denoted μ_β_, within each EDC class in relation to birth weight (g) of 125 male infants in the HOME study, 2003-2006, Cincinnati, OH.

|  | Difference in birthweight (grams) | | | | | |
| --- | --- | --- | --- | --- | --- | --- |
|  | Primary Analysis (GA Included) | | | Secondary Analysis (GA Excluded) | | |
| Independent Variable | Posterior Mean (SD) | 95% CI | 50% CI | Posterior Mean (SD) | 95% CI | 50% CI |
| Phthalate Class and BPA | -3 (17) | (-35, 27) | (-12, 5) | 2(21) | (-37, 42) | (-9, 14) |
| PFAS Class | 1(41) | (-74, 83) | (-12, 5) | -23(45) | (-114, 72) | (-45, -2) |
| PCB Class | 9(8) | (-7, 24) | (4, 13) | 10(11) | (-12, 32) | (4, 17) |
| PDBE Class | -9(28) | (-68, 54) | (-22, 4) | -7(24) | (-57, 39) | (-20, 6) |
| OC Pesticide Class | -20(32) | (-87, 44) | (-37, -3) | 5(48) | (-91, 107) | (-20, 29) |
| DEP | 51(54) | (-57, 158) | (14, 87) | -24(75) | (-170, 126) | (-74, 27) |
| DMP | -88(54) | (-193, 17) | (-125, -52) | -112(76) | (-261, 34) | (-162, -60) |
| Lead | -94(56) | (-203, 23) | (-131, -57) | 34(79) | (-198, 121) | (-85, 20) |
| Mercury | -9(54) | (-112, 97) | (-45, 27) | 15(76) | (-135, 160) | (-35, 67) |
| Race (Black/Other) | 110(175) | (-228, 457) | (-11, 226) | -265(245) | (-788, 212) | (-425, -98) |
| Age at Delivery | 3(15) | (-26, 33) | (-7, 13) | -10(20) | (-47, 30) | (-23, 3) |
| Did not finish high school | -205(154) | (-228, 457) | (-11, 226) | -129(199) | (-526, 268) | (-259, -7) |
| Cotinine (ng/ml >3) | -97(65) | (-221, 28) | (-141, -53) | -169(91) | (-348, 10) | (-229, -108) |
| Low income(<$25,000/yr) | 64(182) | (-289, 416) | (-59, 190) | 282(251) | (-222, 770) | (117, 452) |
| Unemployment | -72(137) | (-348, 194) | (-163, 19) | 311(179) | (-37, 662) | (190, 435) |
| Public/No Insurance | -79(189) | (-454, 289) | (-206, 51) | -5(268) | (-567, 507) | (-180, 175) |
| Marital Status (Unmarried) | -144 (191) | (-529, 234) | (-270, -17) | -157(266) | (-674, 366) | (-331, 17) |
| Pre-natal vitamins (Yes) | 181(117) | (409, 52) | (259, 103) | 193(165) | (510, 131) | (307, 79) |
| BMI (kg/m^2^) | 40(9) | (21, 58) | (33, 46) | 33(13) | (4, 59) | (24, 42) |

**Table S2.** Bayesian estimates of the average of the beta coefficients, denoted μ_β_, within each EDC class in relation to birth weight (g) of 147 female infants in the HOME study, 2003-2006, Cincinnati, OH.

|  | Difference in birthweight (grams) | | | | | |
| --- | --- | --- | --- | --- | --- | --- |
|  | Primary Analysis (GA Included) | | | Secondary Analysis (GA Excluded) | | |
| Independent Variable | Posterior Mean (SD) | 95% CI | 50% CI | Posterior Mean (SD) | 95% CI | 50% CI |
| Phthalate Class and BPA | 7(16) | (-28, 41) | (-1, 17) | 5(20) | (-41, 47) | (-4, 15) |
| PFAS Class | -18(26) | (-70, 36) | (-30, -7) | -24(33) | (-88, 48) | (-38, -12) |
| PCB Class | -5(6) | (-16, 8) | (-8, -1) | -6(6) | (-18, 6) | (10, -2) |
| PDBE Class | -1(14) | (-29, 28) | (-8, 6) | -11(19) | (-51, 39) | (-20, -3) |
| OC Pesticide Class | 15(26) | (-43, 62) | (3, 27) | 17(28) | (-36, 71) | (4, 31) |
| DEP | -9(45) | (-98, 81) | (-39, 21) | -40(50) | (-139, 64) | (-70, -10) |
| DMP | -27(47) | (-117, 64) | (-59, 5) | -22(51) | (-126, 80) | (-55, 12) |
| Lead | -8(47) | (-100, 84) | (-39, 24) | 25(55) | (-83, 130) | (-11, 63) |
| Mercury | 12(40) | (-66, 90) | (-16, 39) | -3(46) | (-95, 85) | (-32, 27) |
| Race (Black/Other) | -188(113) | (-408, 31) | (-265, -113) | -274(129) | (-521, -16) | (-356, -188) |
| Age at Delivery | 7(10) | (-12, 26) | (0, 13) | 2(11) | (-20, 24) | (-6, 9) |
| Did not finish high school | -104(103) | (-307, 96) | (-173, -35) | -100(120) | (-331, 137) | (-181, -15) |
| Cotinine (ng/ml >3) | -51(48) | (-145, 45) | (-84, -19) | -49(58) | (-165, 56) | (-88, -3) |
| Low income(<$25,000/yr) | -61(124) | (-304, 182) | (-144, 23) | -7(158) | (-364, 311) | (-108, 99) |
| Unemployment | -198(94) | (-385, -14) | (-263, -132) | -237(112) | (-455, -24) | (-311, -154) |
| Public/No Insurance | 50(140) | (-213, 344) | (-45, 141) | -9(154) | (-308, 299) | (-111, 93) |
| Marital Status (Unmarried) | -38(130) | (-285, 215) | (-125, 49) | -48(153) | (-319, 259) | (-157, 57) |
| Pre-natal vitamins (Yes) | 118(91) | (297, 60) | (179, 57) | 14(105) | (225, 191) | (88.7, 57) |
| BMI (kg/m^2^) | 12(6) | (1, 25) | (9, 17) | 23(7) | (10, 36) | (18, 27) |


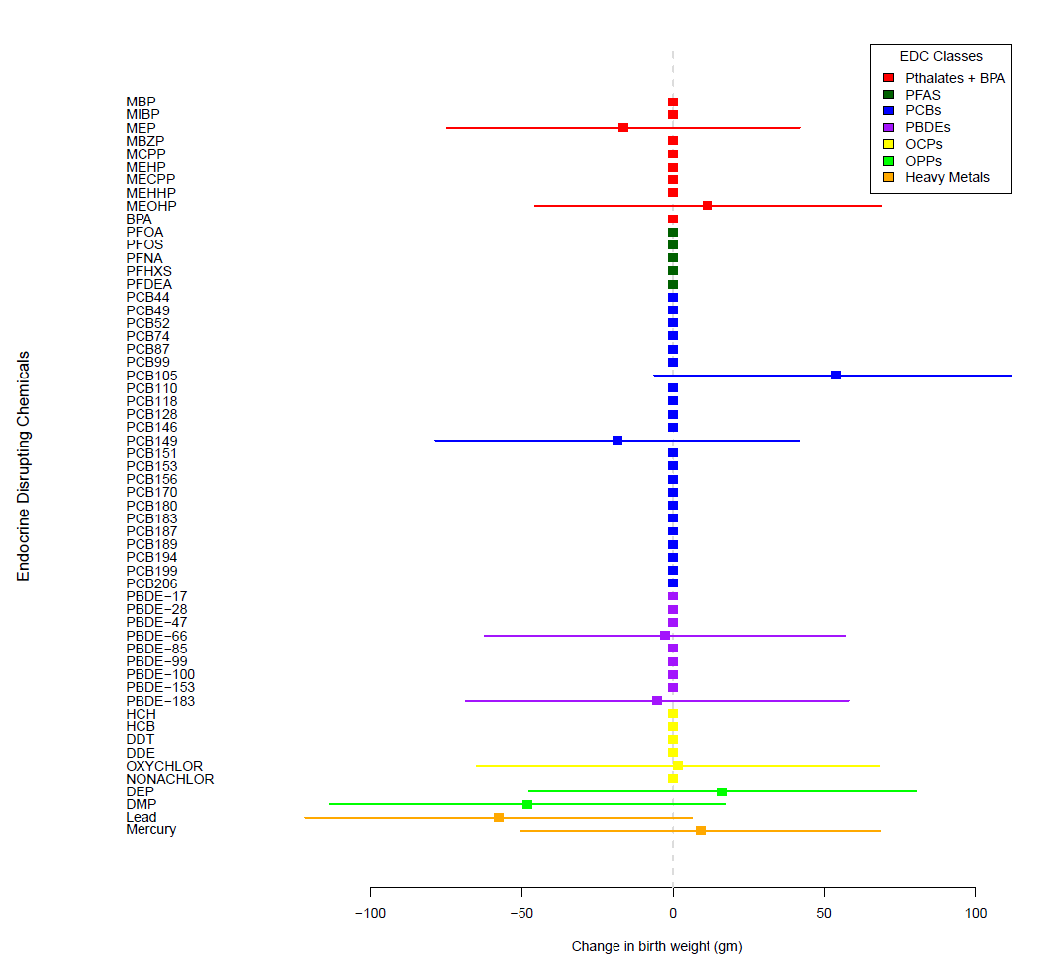


**Figure S3.** 95% CIs for beta coefficients estimated LASSO coefficients, HOME Study, 2003-2006, n=272, Cincinnati, OH. Predictor variables that were dropped from the model by LASSO are denoted by point estimates at zero.

**Figure S4.** 95% CIs for beta coefficients estimated Elastic Net coefficients, HOME Study, 2003-2006, n=272, Cincinnati, OH. Predictor variables that were dropped from the model by Elastic Net are denoted by point estimates at zero.
